# Pedicle Temporalis Fascial Flap with Axial Scalp Flap Obviates Need of Free Flap in Extensive Scalp Wound

**DOI:** 10.1155/2017/4821329

**Published:** 2017-01-17

**Authors:** F. W. Nangole, S. O. Khainga

**Affiliations:** Department of Surgery, University of Nairobi, P.O. Box 30197, Nairobi, Kenya

## Abstract

Extensive scalp defect with exposed bone is best reconstructed with flaps. Majority of these wounds are now routinely reconstructed with free flaps in many centers. Free flaps however require lengthy operative time and may not be available to all patients, where possible less extensive options should thus be encouraged. A sixty-eight-year-old patient presented to us with a Marjolin's ulcer on the vertex of the scalp. After wide local excision a defect of about 17 cm and 12 cm was left. The defect was successfully covered with a combination of an ipsilateral pedicle temporalis fascial flap and an axial supraorbital scalp flap with good outcome. In conclusion wide defects of the scalp can be fully covered with a combination of local flaps. The axial scalp flap and the pedicle temporalis fascial flap where applicable provide an easy and less demanding option in covering such wounds. These flaps are reliable with good blood supply and have got less donor side morbidity.

## 1. Introduction

Scalp wounds require prompt management to prevent morbidities and reduce hospital stay. Reconstruction options for such defects include primary wound closure, healing by secondary intention, skin grafts, and flaps. Scalp defects with exposed bone are best reconstructed with flaps. Small defects are routinely repaired with local scalp flaps as either advancement flaps or transposition or rotational flaps [1–4]. Extensive defects on the other hand are mainly reconstructed with free flaps [5–7].

Among the free flaps routinely utilized are the latissimus dorsi muscle flap, anterior lateral thigh flap, and the radial forearm flap [4–7]. These flaps are able to provide adequate soft tissue that can be utilized to cover extensive scalp defects. Free flaps however have disadvantages. This includes donor side morbidity, prolonged operation time, and need for perioperative monitoring. Free flaps also require patients who are fit to undergo prolonged anesthetic time.

We present a patient with an extensive scalp wound that we successfully managed with multiple local flaps, thus obviating the need for free flaps.

## 2. Case Presentation

**Figure 1:**
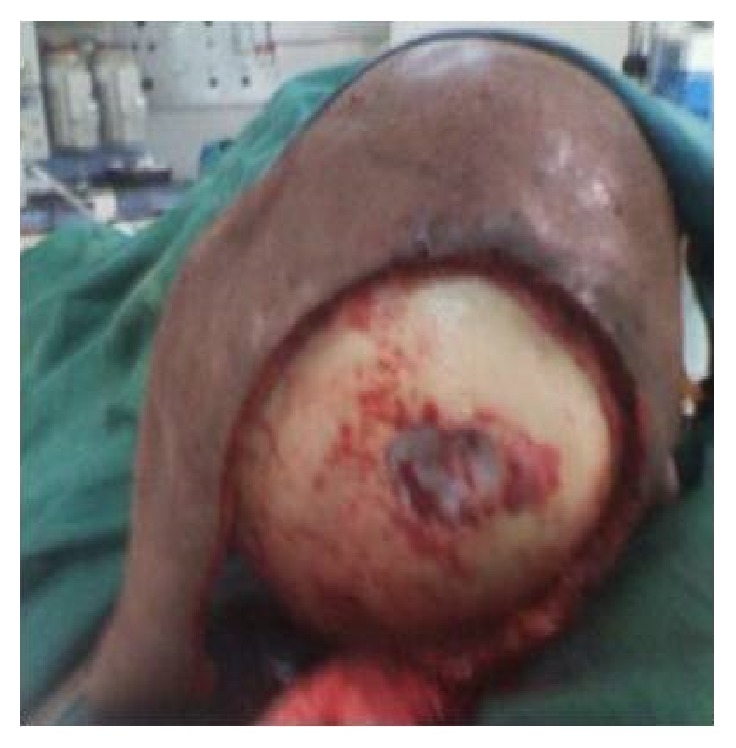
Scalp defect after excision of the tumour.

**Figure 2:**
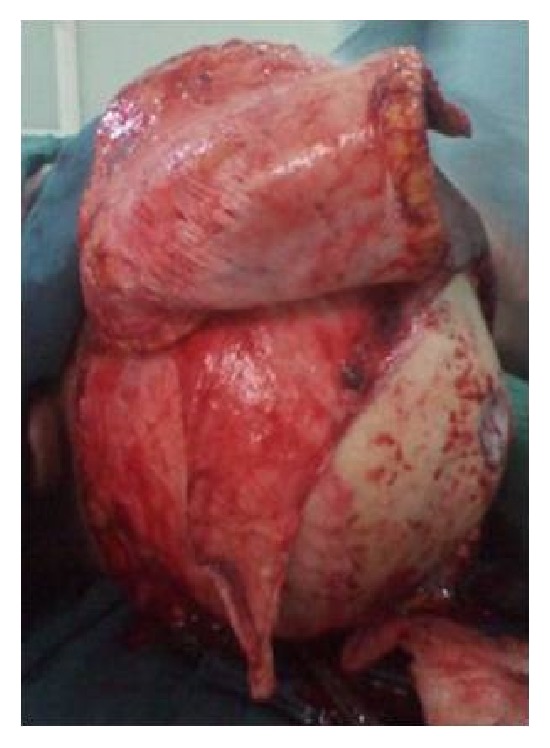
Scalp axial flap raised based on the supraorbital and supratrochlear vessel.

**Figure 3:**
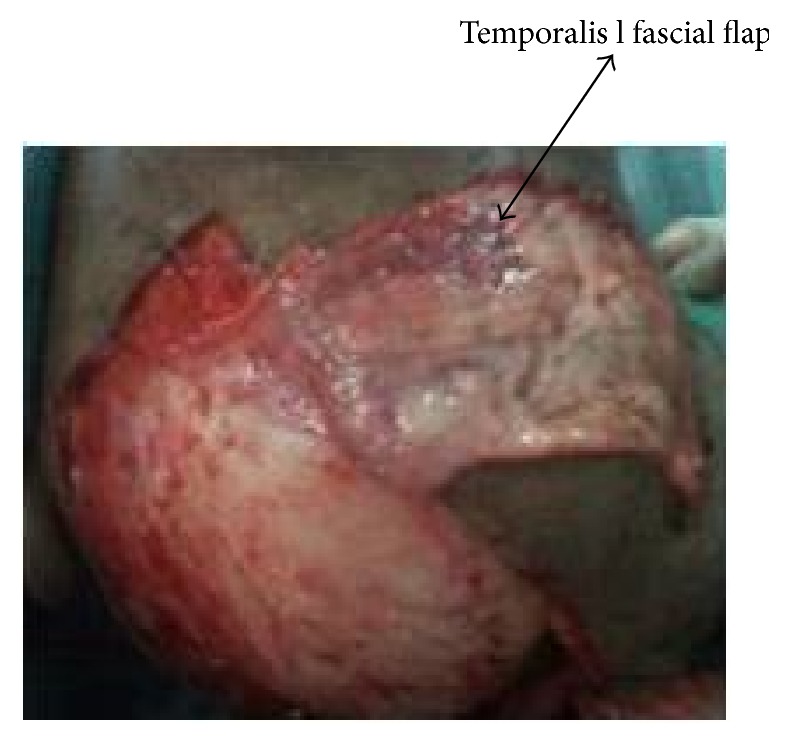
Temporoparietal fascial flap raised on the superficial temporal vessels.

**Figure 4:**
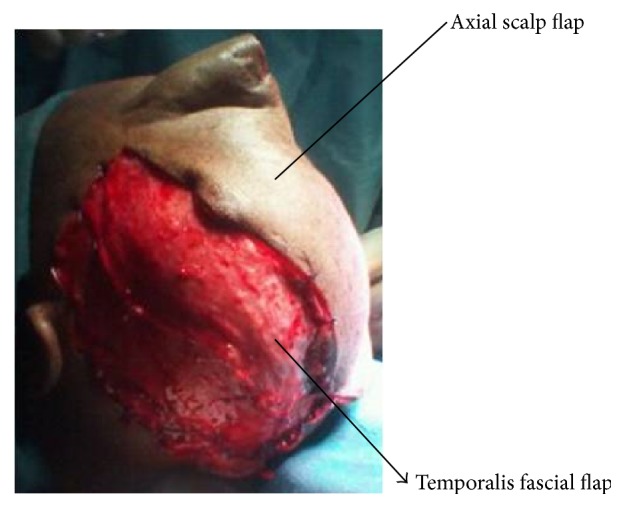
Temporoparietal flap and the axial scalp flap utilized to cover the exposed bone.

**Figure 5:**
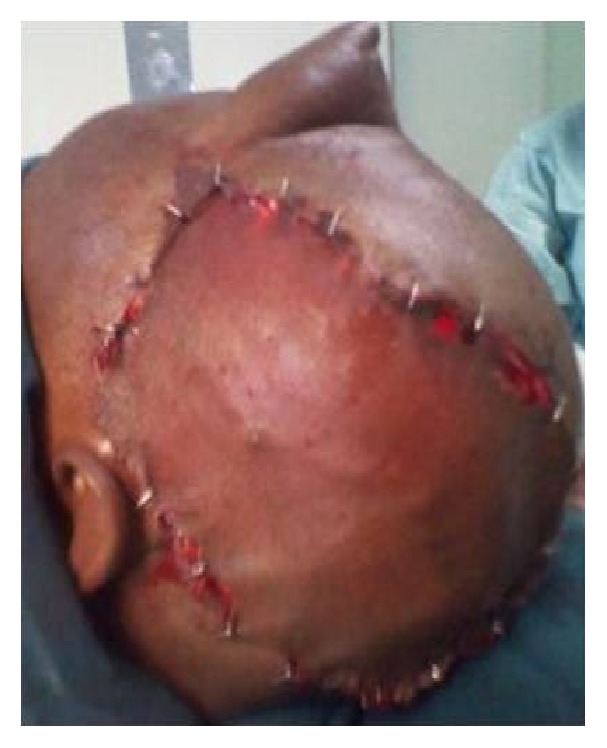
Skin graft used to cover the temporoparietal flap and the temporalis muscle.

A sixty-eight-year-old female patient presented to the plastic surgical clinic at the Kenyatta National Hospital with a Marjolin's ulcer of the scalp. Incisional biopsy revealed squamous cell carcinoma. Radiological investigations done included CT scan and MRI. They revealed a localized lesion with no intracranial involvement. Operatively the patient had wide local excision of the tumour resulting in a scalp defect that measured about 17 cm by 12 cm in size with exposed bone (Figure 1). A scalp axial fasciocutaneous flap raised on the supraorbital/supratrochlear vessels was then fashioned. The flap was raised in the loose areolar plane above the pericranium (Figure 2). The flap was then transposed into the defect though it covered only about two-thirds of the defect. An ipsilateral temporalis fascial flap raised with the superficial temporal artery was then carried out. This was then transposed into the remaining part of the defect (Figures 3 and 4). Split thickness skin graft was then utilized to cover temporalis fascial flap and the donor site for the scalp flap (Figure 5). Postoperatively the flaps did well and the patient did not have any complications. Histology results revealed complete excision of the tumour.

## 3. Discussion

Our patient had an extensive defect that could otherwise best be covered with a free flap. She was however elderly and frail and could possibly not withstand long operative time associated with free flaps. The other option could be to use dermal skin substitutes such as Integra or Alloderm and cover with skin graft after granulation [8, 9]. These substitutes are not only expensive but also unavailable in many developing countries like ours.

The two-flap technique was thus probably the best option for this patient.

The axial scalp flap based on the supraorbital vessels or any other vessel allows for a big chunk of scalp tissue to be mobilized and either rotated or transposed to cover defects. This flap in many instances is able to cover most of the small or moderate scalp defects [3]. It is reliable and the donor site like in our patient is covered by the skin graft. On its own however it may be difficult to cover extensive defects of more than 100 cm^2^. The superficial temporal flap is also a reliable flap based on the superficial temporal vessels. The flap has been widely used either as a pedicle flap or as a free flap [2]. As a pedicle flap it is ideal in covering defects around the frontal and temporal area of the scalp [2].

A combination of these two flaps can thus be utilized to cover extensive scalp defect involving the frontal, temporal, and vertex aspect of the scalp. One however has to be careful when raising the scalp flap so as not to damage the temporal fascial or the superficial temporal blood vessels. Injury to this vessel may make the temporoparietal fascial flap unreliable and it may thus not survive. The pericranium and the temporalis muscle must also be left intact while raising the temporoparietal fascial flap so as to allow for skin grafting of the donor site.

In conclusion though free flaps are now considered the gold standard in covering extensive defects of the flaps, a combination of local flaps can obviate the need of free flap. The temporal parietal fascia flap in combination with the scalp flap may allow one to cover extensive defects involving the temporal and frontal aspect of the scalp. These flaps are reliable and easy to raise and have low donor site morbidity. They are also suited in patients who may not withstand long operative times.
